# Eating disorders in Australia: a commentary on the need to address stigma

**DOI:** 10.1186/s40337-020-00287-3

**Published:** 2020-03-30

**Authors:** Rachel Baffsky

**Affiliations:** The School of Public Health and Community Medicine, The University of New South Wales, UNSW, Sydney, 2052 Australia

**Keywords:** Eating disorders, Stigma, Shame, Media, Health promotion, Popular press, Stereotyping, Labelling, Regulations, Code of conduct

## Abstract

This commentary justifies the need for the Australian government to address stigma and shame in its effort to increase help-seeking by individuals with eating disorders from the intersectional perspective of a health consumer with a history of anorexia nervosa and a public health researcher. It does so in response to the government’s planned 2019 investment of $110 million to subsidise eating disorders treatment services. The commentary identifies stigma and shame as the leading barrier to help-seeking among individuals with eating disorders. It then uses peer-reviewed evidence and analyses of popular press articles to show how media create stigma and shame through labelling and stereotyping individuals with eating disorders in a way that incites status loss and discrimination. The commentary justifies why Australia provides an interesting test case to trial the use of media regulations to address this international problem. It is recommended that the Australian government work with individuals with eating disorders to co-design a Mandatory Code of Conduct to guide media towards a more medicalised approach to representing the diverse spectrum of real individuals who experience eating disorders. This commentary is relevant to an international audience as it provides solutions to common challenges with media representations of individuals with eating disorders found in Western and Eastern contexts.

The need to increase help-seeking among individuals with eating disorders is particularly relevant in the Australian context. As of November 2019, the Australian government will invest $110 million over 4 years to subsidise eating disorder treatment [1]. This has the potential to improve recovery for the 16% of Australians estimated to have a DSM-5 eating disorder [2]. A systematic review of the literature on the perceived barriers towards help-seeking for people with eating disorders found that stigma and shame was more frequently identified as a barrier for accessing treatment than practical factors such as costs and transportation [3]. Stigma is defined as the cooccurrence of “labelling, stereotyping, separation, status loss and discrimination” in a “power situation that allows them” [4]. The purpose of this commentary is to explain how media create stigma and shame for people with eating disorders and justify why media regulations are an appropriate strategy to increase help-seeking.

There is evidence to suggest that media create stigma for people with eating disorders by using its power over public perception to reinforce stereotypes [5–7]. Content analyses of newspapers in the UK, US and China found that articles in all three contexts stereotyped the typical person with an eating disorder as young and female [5–7]. This devalues the experience of men and older people who experience these conditions which creates stigma [5, 6]. Eating disorder articles were also overly focused on the social aetiology of the conditions, even though clinicians and researchers are now more concerned with the biological aetiology [6, 7]. This reinforces the harmful stereotype that eating disorders are easy to recover from as they are a choice, which again creates stigma [5–7].

The way in which the popular press stigmatises individuals with eating disorders through labelling them with undesirable characteristics [4] will now be exemplified through analysis of two real-world articles. The popular press is defined by Sheppard and Seale [7] as a news source with a tabloid agenda. In one distastefully titled article, “The Hunger Games”, the NY Daily News posted photos of celebrities perceived to have anorexia nervosa, highlighting parts of their body that looked “scary skinny”, for example Renee Zellweger’s “freakishly toned back” [8]. This provides a familiar example of how media use derogatory language to devalue celebrities perceived to have eating disorders, creating stigma.

There is evidence to suggest real people with eating disorders feel devalued by the public. A 2016 study of 19 females undergoing inpatient treatment for anorexia nervosa in Canada found that 100% of participants used derogatory language to describe how they believed their illness was perceived by the public [9]. They also reported concealing their eating disorder for fear of stigmatization [9]. Given media create status loss for people with anorexia nervosa by positioning them as victims to the general public [6, 7, 10], and stigma is a barrier to help-seeking, it can be deduced that media is an indirect barrier to help-seeking among people with eating disorders.

Further evidence of media creating stigma for individuals with eating disorders among the general public was found in a 2016 study by Iles and colleagues [11]. This study found that healthy participants who were presented with a stigmatising message about eating disorders perceived individuals with eating disorders as less warm and competent compared to a control group who received a neutral message and this led to greater feelings of contempt [11]. The stigmatising message was entitled, “don’t die for diet” and intentionally evoked the negative stereotype that eating disorders are a lifestyle choice, that is commonly perpetuated by media p. 481 [6, 7, 11]. The Iles and colleagues study provides important learning about how stigmatising media portrayals can lead to the general public holding negative attitudes towards individuals with eating disorders, a problem that was identified as a barrier to help-seeking in the Dimitropoulos and colleagues [9] study.

Stigmatising media messages can also be seen through the way in which the popular press have turned diagnosing celebrities perceived to have anorexia nervosa into a game. This was evident in a Fox News article titled “Skinny or Curvy?” which showed photographs of celebrities pre and post weight loss and encouraged viewers to decide whether they’ve “taken it too far” [12]. Articles like this dismiss the medical nature of the illness and reinforce the stereotype that anorexia nervosa is a lifestyle choice [9].

There is evidence to suggest media should use more medicalised language to describe eating disorders as a strategy to reduce blame-based stigma [5–7]. According to Weiner, Perry and Magnusson’s attribution theory, people are more likely to blame an individual for their disorder if they believe it’s caused by social factors [13]. Wingfield and colleagues [14] found that undergraduate psychology students rated fictional characters with eating disorders as more responsible for their condition when their aetiology was framed as social rather than biological. Crisafulli [15] found similar findings using a sample of undergraduate nursing students. It is concerning that psychology and nursing students displayed blame-based stigma when presented with social aetiologies of eating disorders as they are the future clinicians responsible for treatment. These findings suggest addressing media-induced stigma needs to be a priority for improving the quality of care for eating disorders.

The National Eating Disorders Collaboration (NEDC), Australia’s government-funded collaboration of experts in eating disorders, has suggested that media standards and regulations could be used to counter blame-based stigma towards individuals with eating disorders [16]. To the extent of my knowledge, no country has successfully introduced media regulations to change public attitudes and knowledge towards eating disorders. However, Australia provides an excellent test case to trial this approach as the Australian government has already implemented a Voluntary Industry Code of Conduct to promote more positive body imaging in fashion, media and advertising industries. In 2010 (1 year after the code was implemented), Boyd and Moncrieff-Boyd conducted a content analysis of popular Australian magazines and found that only one out of seven magazines had successfully adhered to the code [17]. Researchers and policy advisors hypothesised that the code has not yet been successful because it is voluntary and have recommended that the Australian government mandate the code through legislation [18–20]. Krawitz [18] argued that since Australia successfully used cigarette advertising legislation to reduce smoking, Australia could feasibly reduce body image issues by mandating the Industry Code of Conduct. This commentary extends Krawitz’s theory and recommends that the Australian government use legislation to regulate media coverage of people with eating disorders in order to reduce stigma and shame and improve help-seeking [18].

It is recommended that Australia develops a Mandatory Industry Code of Conduct that specifically guides media towards a more medicalised approach to reporting individuals with eating disorders. In light of the evidence above, this commentary recommends the inclusion of four codes. First, a code that mandates a more demographically diverse representation of real individuals with eating disorders, as recommended by O’Hara [6]. Second, a code that mandates eating disorder articles to address biological aetiology. Third, a code that prohibits journalists from using derogatory language to label the symptoms of individuals with eating disorders. Fourth, a code that mandates realistic reporting of recovery time [6, 7]. Given the commonality of challenges with media coverage of eating disorders in US, UK and China [5–7] and the globalised nature of digital media, these recommendations have the potential to be generalised internationally.

The author acknowledges several limitations with the Mandatory Industry Code of Conduct approach. Firstly, it has been criticised as being paternalistic [21]. This is potentially problematic as individuals with anorexia nervosa perceive a need for control as a key cause of their eating disorder [22]. For this reason, it is critical that the codes be developed with extensive input from individuals with eating disorders to increase perceived control among this vulnerable group. Secondly, media emphasis on biological aetiology could potentially encourage the public to perceive an individuals’ eating disorder as a helpless condition, because it has biological/genetic origins. For this reason, the fourth code was strategically introduced, to ensure journalists accurately report that individuals with eating disorders can and do recover if they seek help [23].

## Conclusion

This commentary has provided evidence to suggest that unregulated media has contributed to stigma and shame for individuals with eating disorders. Given the strength of this evidence, it is argued that the advantages of introducing media regulations outweigh the disadvantages. This commentary has recommended Australia develop a Mandatory Industry Code of Conduct to guide media towards using more neutral, medical language to depict real people with lived experience of eating disorders from diverse sociodemographic backgrounds. From a practical standpoint, the Mandatory Industry Code of Conduct should be co-designed with individuals with lived experience of eating disorders as they are the most informed about how negligent media coverage perpetuates feelings of stigma and shame. It is recommended the NEDC oversees this process as they are the network of Australia’s experts in the field. Australia’s approach of using a Mandatory Industry Code of Conduct to reduce stigma and shame has the potential to be adopted by other countries if it is proven effective in increasing help-seeking.

## Data Availability

N/A

## Abbreviation

NEDC: National Eating Disorders Collaboration

## References

[1] Aubusson K, Thompson A (2018). The Sydney morning herald. People with eating disorders to get more Medicare-funded treatment.

[2] Hay P, Girosi F, Mond J (2015). Prevalence and sociodemographic correlates of DSM-5 eating disorders in the Australian population. J Eat Disord.

[3] Ali K, Farrer L, Fassnacht DB, Gulliver A, Bauer S, Griffiths KM (2017). Perceived barriers and facilitators towards help-seeking for eating disorders: a systematic review. Int J Eat Disord.

[4] Link BG, Phelan JC (2001). Conceptualizing stigma. Annu Rev Sociol.

[5] Sun Shaojing, He Jinbo, Fan Xitao, Chen Yibei, Lu Xueke (2019). Chinese media coverage of eating disorders: Disorder representations and patient profiles. International Journal of Eating Disorders.

[6] O’Hara SK, Smith KC (2007). Presentation of eating disorders in the news media: what are the implications for patient diagnosis and treatment?. Patient Educ Couns.

[7] Shepherd E, Seale C, Eli K, Ulijaszek S (2016). Eating disorders in the media: the changing nature of UK newspaper reports 1. Obesity, eating disorders and the media.

[8] Daily News (2012). The hunger games: scary-skinny celebrities.

[9] Dimitropoulos G, Freeman VE, Muskat S, Domingo A, McCallum L (2016). “You don’t have anorexia, you just want to look like a celebrity”: perceived stigma in individuals with anorexia nervosa. J Ment Health.

[10] Saguy AC, Gruys KJ (2010). Morality and health: news media constructions of overweight and eating disorders. Soc Probl.

[11] Iles IA, Seate AA, Waks LJ (2016). Eating disorder public service announcements: analyzing effects from an intergroup affect and stereotype perspective. Health Educ J.

[12] Fox News (2016). Celebrity scale: skinny or curvy?.

[13] Weiner B, Perry RP, Magnusson J (1988). An attributional analysis of reactions to stigmas. J Pers Soc Psychol.

[14] Wingfield N, Kelly N, Serdar K, Shivy VA, Mazzeo SE (2011). College students’ perceptions of individuals with anorexia and bulimia nervosa. Int J Eat Disord.

[15] Crisafulli MA, Von Holle A, Bulik CM (2008). Attitudes towards anorexia nervosa: the impact of framing on blame and stigma. Int J Eat Disord.

[16] National Eating Disorders Collaboration. Barriers to care. https://www.nedc.com.au/eating-disorders/treatment-and-recovery/barriers-to-care/. Accessed 3 May 2019.

[17] Boyd ER, Moncrieff-Boyd J (2011). Swimsuit issues: promoting positive body image in young women’s magazines. Health Promot J Austr.

[18] Krawitz M (2014). Beauty is only photoshop deep: legislating models’ BMIs and photoshopping images. J Law Med.

[19] Zubcevic-Basic NJ (2012). The conversation. Reining in advertisers to curb Australia’s body image distortion.

[20] Mamamia FM (2011). Voluntary code of conduct: why I was wrong.

[21] Gladstone J (2016). The skinny on BMI-based hiring: an assessment of the legality and effectiveness of Israel's weight restriction law. Wash U Global Stud L Rev.

[22] Salafia EHB, Jones ME, Haugen EC, Schaefer MK (2015). Perceptions of the causes of eating disorders: a comparison of individuals with and without eating disorders. J Eat Disord.

[23] The Butterfly Foundation. A strategic communication framework. In: Media guidelines: The National Eating Disorders Collaboration; 2010. http://nedc.dev.blissmedia.com.au/assets/NEDC-Publications/NEDC-Strategic-Communication-Framework-Final.pdf. Accessed 3 Dec 2019.

